# On Cancer

**Published:** 1817-04

**Authors:** Charles Aldis


*For the London Medical and Physical Journal.*

### *On Cancer;* by Charles Aldis, Esq.

IN answer to a paper inserted in the last Number of your Journal, under the signature of "A Valetudinarian," I can only reply, that it would indeed be desirable if that gentleman could throw any information which might "lead to a more accurate discrimination of the diseases so generally confounded under the term Cancer; but I much question his ability so to do, believing firmly that those who have paid great attention to it know little or nothing of the requisites whereby real cancer can be judged; and I think it would be at best but arguing *(de lanâ caprina)* to attempt a description. You will not infer from this, that I am an advocate for inattention as to the nature and cause of disease, any further than to those complaints of which we shall, from their *very nature*, always remain ignorant, and which I believe to be the case by a vast majority, even with those who are well acquainted with a method of cure.

I was led to this remark from a conviction that one of the modes of treatment, *id est*, by *extraction*, alluded to myself, a plan by which I have for several years been successful, as far as relates to the cure of this disease; and that I should say with you, Mr. Editor, that I should feel myself much obliged if that gentleman would pursue his remarks systematically.

*Nelson-square, Blackfriars Road;*

*March* 3, 1817.

